# Accelerated hyper-versus normofractionated radiochemotherapy with temozolomide in patients with glioblastoma: a multicenter retrospective analysis

**DOI:** 10.1007/s11060-021-03926-0

**Published:** 2021-12-23

**Authors:** Rainer J. Klement, Ilinca Popp, David Kaul, Felix Ehret, Anca L. Grosu, Bülent Polat, Reinhart A. Sweeney, Victor Lewitzki

**Affiliations:** 1 Klinik für Strahlentherapie, Leopoldina Krankenhaus Schweinfurt, MVZ Leopoldina Krankenhaus, Robert-Koch-Straße 10, 97422 Schweinfurt, Germany; 2 Klinik für Radio-Onkologie, Universitätsspital Zürich, Universität Zürich, 8006 Zurich, Switzerland; 3 Klinik für Strahlenheilkunde, Universitätsklinikum Freiburg, 79106 Freiburg, Germany; 4 Klinik Für Radioonkologie und Strahlentherapie, Charité - Universitätsmedizin Berlin, 13353 Berlin, Germany; 5 German Cancer Consortium (DKTK), partner site Berlin, Berlin, Germany; 6 Berlin Institute of Health at Charité – Universitätsmedizin Berlin, 10117 Berlin, Germany; 7 German Cancer Consortium (DKTK), Partner Site Freiburg, German Cancer Research Center (DKFZ), Heidelberg, Germany; 8 Klinik für Strahlentherapie und Radioonkologie, Universitätsklinikum Würzburg, Josef-Schneider-Straße 11, 97080 Würzburg, Germany

**Keywords:** Accelerated hyperfractionation, Altered fractionation, Glioblastoma, Radiotherapy, Temozolomide

## Abstract

**Background and purpose:**

The standard treatment of glioblastoma patients consists of surgery followed by normofractionated radiotherapy (NFRT) with concomitant and adjuvant temozolomide chemotherapy. Whether accelerated hyperfractionated radiotherapy (HFRT) yields comparable results to NFRT in combination with temozolomide has only sparsely been investigated. The objective of this study was to compare NFRT with HFRT in a multicenter analysis.

**Materials and methods:**

A total of 484 glioblastoma patients from four centers were retrospectively pooled and analyzed. Three-hundred-ten and 174 patients had been treated with NFRT (30 × 1.8 Gy or 30 × 2 Gy) and HFRT (37 × 1.6 Gy or 30 × 1.8 Gy twice/day), respectively. The primary outcome of interest was overall survival (OS) which was correlated with patient-, tumor- and treatment-related variables via univariable and multivariable Cox frailty models. For multivariable modeling, missing covariates were imputed using multiple imputation by chained equations, and a sensitivity analysis was performed on the complete-cases-only dataset.

**Results:**

After a median follow-up of 15.7 months (range 0.8–88.6 months), median OS was 16.9 months (15.0–18.7 months) in the NFRT group and 14.9 months (13.2–17.3 months) in the HFRT group (p = 0.26). In multivariable frailty regression, better performance status, gross-total versus not gross-total resection, *MGMT* hypermethylation, *IDH* mutation, smaller planning target volume and salvage therapy were significantly associated with longer OS (all p < 0.01). Treatment differences (HFRT versus NFRT) had no significant effect on OS in either univariable or multivariable analysis.

**Conclusions:**

Since HFRT with temozolomide was not associated with worse OS, we assume HFRT to be a potential option for patients wishing to shorten their treatment time.

## Introduction

Glioblastoma (GB) is the most common malignant tumor of the central nervous system in adults [1], accounting for approximately 69% of all malignant adult brain tumors in Germany [2]. The prognosis remains dismal, with a median overall survival (OS) for the whole patient population below 21 months and 5-year OS rates below 10% after standard-of-care trimodal therapy, consisting of surgery followed by radiotherapy with concomitant and adjuvant temozolomide (TMZ) chemotherapy [3–5].

Prognostic factors for patients with GB are related to patient-, treatment- and tumor characteristics. Patient-related prognostic factors include age at diagnosis, clinical and neurological performance at diagnosis or recurrence [6–9], as well as body mass index [10–12], and blood glucose levels [13–17]. Treatment-related factors include gross total resection, concomitant and adjuvant use of TMZ, tumor-treating fields, and aggressive salvage therapy (if possible) [4, 18–21]. Molecular factors include methylation status of the O^6^-methylguanine-DNA methyltransferase (*MGMT*) promoter [22] and mutation status of isocitrate dehydrogenase 1 and 2 (*IDH 1/2*) [23–25]. The role of telomerase reverse transcriptase (*TERT*) promoter mutation remains ambiguous to this date [26, 27].

The analysis of Brain Tumor Study Group protocols (1966–1975) revealed a radiotherapeutic dose–effect relationship [28], suggesting dose escalation as a viable possibility of treatment optimization. The main focus of accelerated hyperfractionated radiotherapy (HFRT) was to exploit the differences in redistribution, reoxygenation, repopulation, and DNA damage repair between normal and tumor cells [29, 30] on the way to dose escalation.

Several prospective glioma trials from the pre-TMZ era have evaluated the potential of HFRT [31–34]. HFRT with or without dose escalation however failed to show any superiority in OS or progression-free survival (PFS) in comparison to normofractionated radiotherapy (NFRT) [35]. A more recently published randomized controlled trial comparing chemo-radiation protocols of dose escalated HFRT with NFRT did not point towards any benefit of HFRT in terms of OS [36].

In addition to the evidence for non-inferiority of HFRT in the abovementioned trials, HFRT schemes significantly reduce treatment time, even though the absolute number of radiotherapy treatments is higher in NFRT schemes. Therefore, the rationale to use it as an alternative scheme for patients is to shorten the time of irradiation from 6 to less than 4 weeks. Furthermore, radiobiologists have hypothesized a reduction of late radiation injury as well as reduced repopulation rates in tumor cells [37]. The current treatment standard for primary GB is the combination of radiotherapy and TMZ according to the Stupp protocol [38] plus an eventual adjuvant therapy with tumor treating fields which may add a few months to the PFS and OS [4]. To the best of our knowledge, there are no prospective randomized trials investigating the efficacy of HFRT versus NFRT in combination with TMZ, with only a couple of monocentric retrospective studies reporting comparable outcomes [39, 40]. To further explore any differences that might exist between HFRT and NFRT, we performed a retrospective multicenter analysis of GB patients which allowed us to model the effects of both schemes on PFS and OS while accounting for known prognostic factors.

## Materials and methods

This work is based on data of patients with newly diagnosed GB treated from 10/2004 to 7/2018 at four tertiary care institutions. Variables of interest were retrospectively collected by each center and inserted into a Microsoft® Access database, which contained pre-specified selection possibilities for the value assignment of categorical variables. The anonymized center-specific databases were then exported and pooled into a single Microsoft® Excel file, which was further processed with R statistical software.

Inclusion criteria for this analysis were at least one follow-up of OS, age ≥ 18 years and having received either HFRT (37 × 1.6 Gy or 30 × 1.8 Gy twice/day) or NFRT (30 × 1.8 Gy, 30 × 2 Gy or 30 × 1.8 Gy with 2 Gy simultaneously integrated boost). This resulted in 484 patients being eligible for analysis of which 310 had received NFRT and 174 HFRT. Differences between the NFRT and HFRT group were assessed using the Wilcoxon rank sum test and Fisher’s exact test for continuous and categorical variables, respectively. PFS was calculated as the time difference between the start of treatment and first clinical or radiological progression. The latter had to be confirmed according to the Response Assessment in Neuro-Oncology (RANO) criteria [41] by a board-certified neuroradiologist. Follow-up included clinical and radiological evaluation every 3 months or depending on the patients’ performance status. Salvage treatment was defined as any treatment initiated after tumor progression (repeat irradiation, surgery, bevacizumab, TMZ, and combinations thereof).

The primary outcome of interest for this study was OS, with PFS as a secondary outcome, both calculated according to the Kaplan–Meier method. PFS and OS were correlated with patient-, tumor- and treatment-related variables via univariable and multivariable Cox frailty models. The frailty model accounts for clustering of patients within centers and therefore for any unmeasured center-specific factors that may affect the outcome (e.g. patient selection bias) [42, 43]. The Cox frailty model can be written as

$$h_{i} \left( t \right) = h_{0} \left( t \right)\exp \left( {{\varvec{X}}_{i} \beta + \alpha_{j} } \right) = h_{0} \left( t \right)\exp \left( {\alpha_{j} } \right){\text{exp}}\left( {{\varvec{X}}_{i} \beta } \right)$$

where ${{\varvec{X}}}_{i}$ is a vector of covariates for patient $i$, ${h}_{0}\left(t\right)$ the baseline hazard function and ${\alpha }_{j}$ is the random effect associated with the $j$-th cluster (clinic) that acts as a multiplier on the baseline hazard. A log-normal distribution was assumed for the frailty terms, equivalent to a normal distribution of the random effects $\mathrm{exp}\left({\alpha }_{j}\right)$.

For multivariable modelling, we tried to utilize as many variables as possible [44, 45] by imputing the covariates with missing information using the multiple imputation by chained equations R package ‘mice’ [46]. A “missing at random” mechanism was assumed being responsible for missing variables, with all variables given in Table 1 as well as follow-up time and OS being added into the imputation model. Variables were imputed in the order of their number of missing cases. Logistic regression and predictive mean matching were used for imputing binary and continuous variables, respectively. A total of 100 imputation data sets were created. Each was used to fit a Cox frailty regression model, and the final Cox model was obtained by pooling the coefficients of these 100 Cox models together. For sensitivity analysis, a Cox model using only the complete cases (with no missing values) was built and compared to the Cox model resulting from the imputed datasets.

**Table 1:** Baseline characteristics

Variable	Unit	Overall cohort (N = 484)	NFRT (N = 310)	HFRT (N = 174)	p-value
Clinic	Würzburg	149 (30.8%)	39 (12.6%)	110 (63.2%)	< 0.0001*
	Freiburg	135 (27.9%)	135 (43.5%)	0	
	Berlin	129 (26.6%)	67 (21.6%)	62 (35.6%)	
	Schweinfurt	71 (14.7%)	69 (22.3%)	2 (1.2%)	
Age	Years: Median (range)	60 (22–85)	61 (23–85)	59 (22–81)	0.098
Gender	Female	186 (38.4%)	122 (39.4%)	64 (36.8%)	0.627
	Male	298 (61.6%)	188 (60.6%)	110 (63.2%)	
KPS	Median (range)	80 (40–100)	80 (40–100)	80 (40–100)	0.222
	Unknown	2	2	0	
Main tumor localization	Frontal	132 (27.3%)	73 (23.5%)	59 (33.9%)	0.019
	Parietal	89 (18.4%)	51 (16.5%)	38 (21.8%)	
	Temporal	138 (28.5%)	99 (31.9)	39 (22.4%)	
	Multifocal	78 (16.1%)	56 (18.1%)	22 (12.6%)	
	Other	47 (9.7%)	31 (10%)	16 (9.2%)	
Surgery extent	Complete resection	186 (39.7%)	121 (40.6%)	65 (38.0%)	0.088
	Incomplete resection	179 (38.2%)	117 (39.3%)	62 (36.3%)	
	Debulking	25 (5.3%)	19 (6.4%)	6 (3.5%)	
	Biopsy	79 (16.8%)	41 (13.7%)	38 (22.2%)	
	Unknown	15	12	3	
Complete resection	No	283 (60.3%)	177 (59.4%)	106 (62.0%)	0.897
	Yes	186 (39.7%)	121 (40.6%)	65 (38.0%)	
	Unknown	15	12	3	
MGMT hypermethylation	No	189 (54.0%)	125 (54.3%)	64 (53.3%)	0.910
	Yes	161 (46.0%)	105 (45.7%)	56 (46.7%)	
	Unknown	134	80	54	
IDH mutation	No	308 (91.4%)	216 (90.8%)	92 (92.9%)	0.748
	Yes	29 (8.6%)	22 (9.2%)	7 (7.1%)	
	Unknown	147	72	75	
PTV	ccm^3^: Median (range)	272 (31–1576)	264 (31–1576)	293 (61–771)	0.0071*
	Unknown	33	19	14	
Temozolomide	No	30 (6.2%)	25 (8.1%)	5 (2.9%)	0.121
	Simultaneous	46 (9.5%)	27 (8.7%)	19 (10.9%)	
	Simultaneous + sequential	399 (82.4%)	252 (81.3%)	147 (84.5%)	
	Sequential	7 (1.4%)	4 (1.3%)	3 (1.7%)	
	Other	2 (0.4%)	2 (0.6%)	0	
Concomitant steroids	No	170 (37.4%)	132 (44.7%)	38 (23.9%)	< 0.0001*
	Yes	284 (62.6%)	163 (55.3%)	121 (76.1%)	
	Unknown	30	15	15	
Salvage treatment	No	169 (38.8%)	96 (33.4%)	73 (49.0%)	0.0019*
	Yes	267 (61.2%)	191 (66.6%)	76 (51.0%)	
	Unknown	48	23	25	

Differences between the NFRT and HFRT groups were assessed using the Wilcoxon and Fisher’s exact test for continuous and categorical variables, respectively

*p < 0.01 (statistically significant)

Statistical significance was defined as p-values < 0.01. This threshold was chosen based on the conversion between p-values and minimum Bayes factors [47]. Bayes factors (or likelihood ratios in case of simple hypotheses) measure the strength of evidence between two competing hypotheses [48]. In exploratory analyses, a p-value of 0.01 corresponds to a minimum Bayes factor of 1/6.5, providing moderate-strong evidence against the null hypothesis [47]. All analyses were performed within the statistical programming language R version 4.0.3 [49].

## Results

Table 1 shows the baseline characteristics of the 484 patients fulfilling the inclusion criteria for this study. There was a significant difference between the NFRT and HFRT group with respect to the treating institution, because HFRT patients mainly stemmed from two hospitals. Other significant differences between the NFRT and HFRT groups existed for planning target volume (PTV) (larger in the HFRT group), steroid administration (more frequent in the HFRT group), and salvage treatment (less frequent in the HFRT group). The exact form of salvage treatment was known for 266 patients and most frequently consisted of surgery (23.3% of cases), repeat radiotherapy (16.5%), TMZ (16.2%), and the combination of surgery and TMZ (14.3%).

**Fig. 1:**
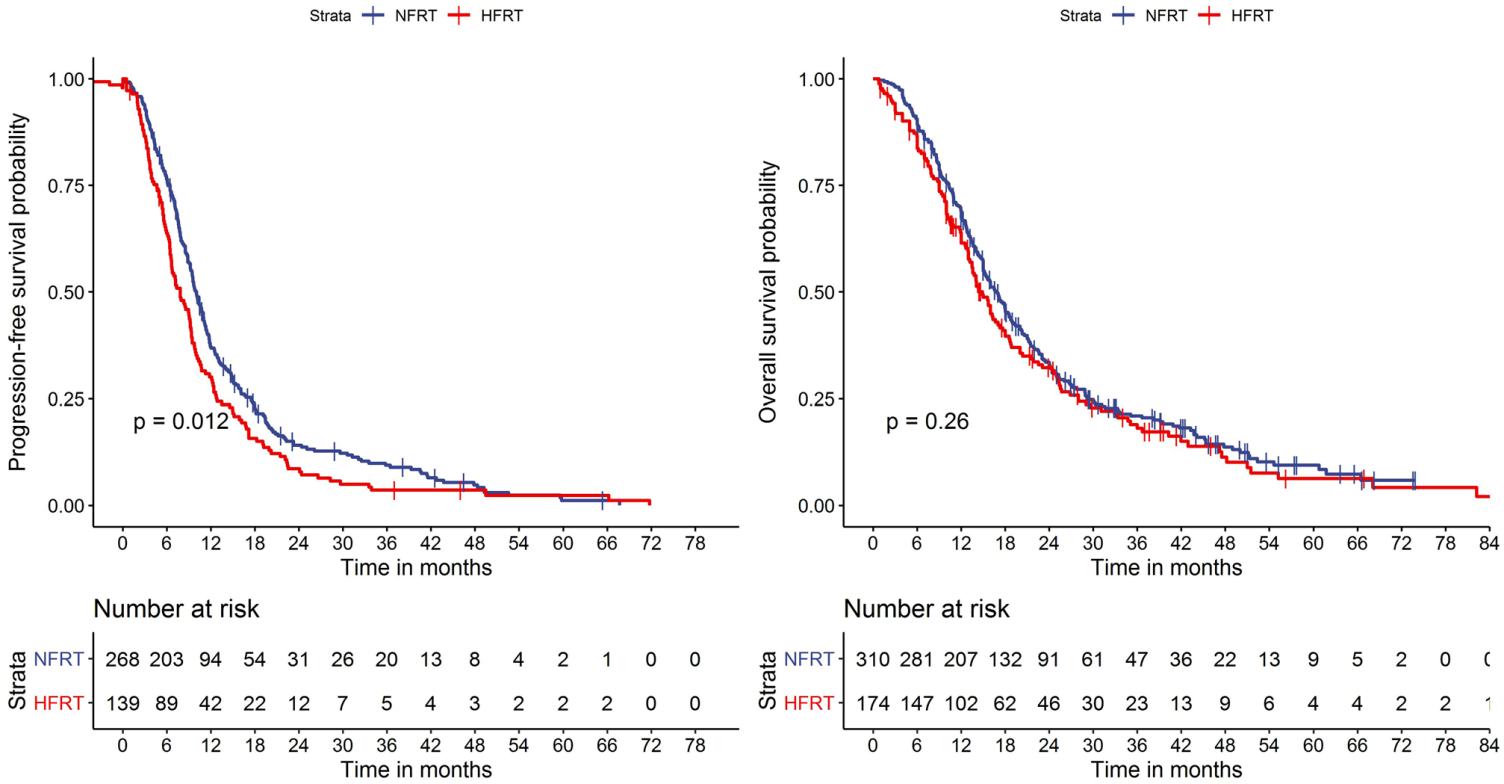
Progression-free and overall survival probability as a function of follow-up time computed with the Kaplan–Meier estimator and stratified according to treatment schedule (statistical significance at p < 0.01). HFRT: Hyperfractionated radiotherapy NFRT: Normofractionated radiotherapy

Kaplan–Meier survival curves stratified according to treatment protocol are shown in Fig. 1. Median follow-up for the whole cohort was 15.7 months (range 0.8–88.6 months). During individual follow-up, tumor progression occurred in 385 out of 410 patients (it was unknown for 74 patients), and 394 of the 484 patients had died. Median PFS was 10.0 months (95% confidence interval [CI] 9.2–11.0) in the NFRT group and 7.9 months (6.6–9.3 months) in the HFRT group, which was almost significantly different (p = 0.012). Median OS was 16.9 months (15.0–18.7 months) in the NFRT group and 14.9 months (13.2–17.3 months) in the HFRT group (p = 0.26). OS was 16.0 months in both groups combined (95% CI 15.0–17.6 months).

A difference in PFS and OS between centers was evident from univariable Cox regression analysis (Table 2). Given that these differences might reflect differences in some unmeasured hospital-specific variables, frailty models were used to fit the other univariable models reported in Table 2. Factors significantly associated with longer PFS were higher KPS, frontal versus multifocal tumor location, gross total resection, *MGMT* hypermethylation, *IDH* mutation and simultaneous plus sequential TMZ versus no TMZ administration. Factors significantly associated with longer OS were younger age, higher KPS, frontal versus multifocal tumor location, gross total resection, MGMT hypermethylation, *IDH* mutation, simultaneous plus sequential TMZ versus no TMZ administration, no steroid administration and salvage treatment.

**Table 2:** Results of the univariable Cox frailty model analyses

Variable	PFS				Overall survival			
	HR	95% CI		p-value	HR	95% CI		p-value
		Lower	Upper			Lower	Upper	
Clinic								0.273
Clinic 2 vs. Clinic 1	1.059	0.813	1.380	0.672	1.155	0.893	1.494	
Clinic 3 vs. Clinic 1	1.658	1.278	2.152	0.0014*	1.925	1.471	2.520	< 0.0001*
Clinic 4 vs. Clinic 1	0.601	0.423	0.854	0.0045*	1.113	0.806	1.537	0.516
RT-protocol: HFRT vs NFRT	1.213	0.933	1.577	0.149	1.258	0.963	1.644	0.093
Age (10 years increase)	1.144	1.036	1.263	0.0078*	1.255	1.143	1.379	< 0.0001*
Gender: Female vs. Male	1.203	0.975	1.483	0.085	1.394	1.131	1.719	0.0019*
KPS	0.980	0.971	0.989	< 0.0001*	0.975	0.967	0.984	< 0.0001*
Main tumor localization								
Parietal vs. Frontal	0.870	0.642	1.179	0.370	0.820	0.606	1.110	0.200
Temporal vs. Frontal	0.840	0.639	1.105	0.213	0.849	0.647	1.114	0.238
Multifocal vs. Frontal	1.567	1.118	2.196	0.0092*	1.713	1.245	2.357	0.00094*
Other vs. Frontal	1.010	0.695	1.467	0.960	1.197	0.830	1.724	0.336
Surgery extent: Gross total vs. not gross total resection ^a^	0.695	0.564	0.858	0.00070*	0.569	0.461	0.703	< 0.0001*
MGMT hypermethylation: Yes vs. No	0.456	0.354	0.587	< 0.0001*	0.426	0.330	0.551	< 0.0001*
IDH mutation: Yes vs. No	0.442	0.263	0.742	0.0020*	0.249	0.132	0.472	< 0.0001*
PTV (100 cm^3^ increase)	1.062	0.992	1.136	0.084	1.131	1.064	1.203	< 0.0001*
Temozolomide								
Simultaneous + sequential vs. none	0.500	0.322	0.777	0.0021*	0.307	0.207	0.455	< 0.0001*
Simultaneous or sequential vs. none	0.787	0.441	1.402	0.416	0.656	0.406	1.060	0.085
Steroids: Yes vs. No	1.375	1.068	1.770	0.014	1.948	1.549	2.450	< 0.0001*
Salvage: Yes vs. No ^b^	–	–	–	–	0.531	0.426	0.663	< 0.0001*

Frailty models accounting for clustering of patients within hospitals were fitted except for the model including clinic as the dependent variable

*CI* confidence interval; *HR* hazard ratio; *PFS* progression-free survival

^a^Not gross total resection comprises incomplete resection, debulking and biopsy

^b^Salvage therapy always provided after tumor progression, therefore no evaluation

After creating 100 imputation datasets, we fitted a separate multivariable Cox frailty model to each of these datasets and pooled the regression coefficients and associated p-values. The average hazard ratios and p-values are given in Table 3. After controlling for many possible confounders, higher KPS, gross total versus not gross total resection, *MGMT* hypermethylation, *IDH* mutation, smaller PTV size and salvage therapy were significantly associated with longer OS (all p < 0.01). RT schedule (HFRT versus NFRT) had no significant effect on OS (p = 0.108). Also, in contrast to univariable analysis, multifocal disease was no longer associated with worse OS. The frailty term modelling heterogeneity between hospitals was on the threshold of statistical significance (p = 0.010), showing the importance of its inclusion into the model.

**Table 3:** Results of the multivariable Cox model analyses on imputed datasets

Variable	Overall survival (N = 484, 394 events)			
	HR	95% CI		p-value
		Lower	Upper	
Age (10 years increase)	1.100	0.989	1.220	0.128
Gender: Female vs. Male	1.240	0.997	1.540	0.036
KPS	0.983	0.974	0.992	0.00010*
Main tumor localization				
Parietal vs. frontal	0.820	0.601	1.120	0.534
Temporal vs. frontal	0.994	0.749	1.320	0.985
Multiple vs. frontal	1.200	0.862	1.680	0.247
Other vs. frontal	1.140	0.777	1.670	0.207
Surgery extent: Gross total vs. not gross total resection^a^	0.641	0.513	0.801	< 0.0001*
MGMT hypermethylation: Yes vs. No	0.415	0.329	0.524	< 0.0001*
IDH mutation: Yes vs. No	0.393	0.247	0.625	< 0.0001*
PTV (100 cm^3^ increase)	1.090	1.020	1.180	0.00095*
RT protocol: HFRT vs. NFRT	1.210	0.916	1.600	0.108
Temozolomide				
Simultaneous and sequential vs. none	0.583	0.380	0.896	0.016
Simultaneous or sequential vs. none	1.120	0.682	1.850	0.528
Steroids: Yes vs. No	1.340	1.040	1.710	0.094
Salvage: Yes vs. No	0.607	0.476	0.776	< 0.0001*
Frailty (clinic)				0.010

Hazard ratios and p-values are pooled estimates from 100 Cox frailty models with a normal distribution for the random effects. Each model had been fitted to one of 100 datasets with slightly different imputations of missing variables (see Materials and Methods for details)

^a^Not gross total resection comprises incomplete resection, debulking and biopsy

*CI* Confidence interval; *HR* Hazard ratio

*p < 0.01 (statistically significant)

A multivariable Cox model fit to the original dataset with complete observations only resulted in similar hazard ratios (Table 4). However, the uncertainties were larger due to the smaller number of observed events (258 patients were not considered due to missing variables), and only MGMT hypermethylation, surgery extent, PTV size, and salvage therapy reached the threshold of statistical significance (p < 0.01).

**Table 4:** Results of the multivariable Cox model analyses on complete observations only

Variable	Overall survival (N = 226, 177 events)			
	HR	95% CI		p-value
		Lower	Upper	
Age (10 years increase)	1.143	0.970	1.347	0.112
Gender: Female vs. Male	1.367	0.971	1.925	0.073
KPS	0.994	0.981	1.008	0.412
Main tumor localization				
Parietal vs. frontal	1.101	0.667	1.817	0.706
Temporal vs. frontal	1.281	0.749	1.320	0.265
Multifocal vs. frontal	0.978	0.593	1.614	0.932
Other vs. frontal	1.585	0.854	2.942	0.144
Surgery extent: Gross total vs. not gross total resection^a^	0.554	0.394	0.778	0.00064*
MGMT hypermethylation: Yes vs. No	0.429	0.304	0.605	< 0.0001*
IDH mutation: Yes vs. No	0.320	0.124	0.824	0.018
PTV (100 cm^3^ increase)	1.281	1.110	1.493	0.0015*
RT protocol: HFRT vs. NFRT	1.019	0.653	1.589	0.935
Temozolomide				
Simultaneous and sequential vs. none	0.660	0.333	1.308	0.234
Simultaneous or sequential vs. none	2.076	0.958	4.499	0.064
Steroids: Yes vs. No	1.298	0.888	1.899	0.178
Salvage: Yes vs. No	0.452	0.312	0.656	< 0.0001*
Frailty (clinic)				0.029

*CI* Confidence interval; *HR* Hazard ratio; *PFS* Progression-free survival

^a^Not gross total resection comprises incomplete resection, debulking and biopsy

*Statistically significant (p < 0.01)

## Discussion

The main aim of this study was to compare patients who had received HFRT with those having received NFRT with respect to PFS and OS. The strength of this analysis is that patients were pooled from four different hospitals, resulting in a large cohort of 484 patients for which several putatively prognostic factors were known. The analysis indicates that HFRT yielded comparable outcomes to NFRT.

This confirms previous findings that accelerated HFRT achieves treatment effects comparable to NFRT in a shorter time frame [39, 40]. The lower α/β ratio of normal brain tissue for late reactions, for which an α/β value of 2 Gy is widely accepted, favors a hyperfractionated acceleration instead of a hypofractionated one. The biologically effective dose (BED) inside the tumor of HFRT protocols may vary considerably because of uncertainties about the kick-off time when repopulation sets in, ${T}_{k}$, and the doubling time ${T}_{d}$ of repopulation. Radiobiological estimates for GB resulted in ${T}_{k}$ = 37 days and ${T}_{d}$ = 15.4 days, indicating a negligible influence of fractionation, but these estimates were obtained based on PFS rather than on the more reliable endpoint OS [50]. Using the formula provided by Lee et al. [51]

$${\text{BED}} = \left\{ \begin{gathered} nd\left( {1 + \frac{d}{{{\raise0.7ex\hbox{$\alpha $} \!\mathord{\left/ {\vphantom {\alpha \beta }}\right.\kern-\nulldelimiterspace} \!\lower0.7ex\hbox{$\beta $}}}}} \right),\quad T \le T_{k} \hfill \\ nd\left( {1 + \frac{d}{{{\raise0.7ex\hbox{$\alpha $} \!\mathord{\left/ {\vphantom {\alpha \beta }}\right.\kern-\nulldelimiterspace} \!\lower0.7ex\hbox{$\beta $}}}}} \right) - \frac{{\ln 2 \left( {T - T_{k} } \right)}}{{\alpha \cdot T_{d} }},\quad T > T_{k} \hfill \\ \end{gathered} \right.$$

and adopting $\mathrm{\alpha }/\upbeta$ = 8 Gy and $\mathrm{\alpha }$=0.12 Gy^−1^ for GB [50], we calculated BEDs for the different schemes as follows:

- 30 × 1.8 Gy twice/day ($T$=21 days): BED = 66.2 Gy;
- 37 × 1.6 Gy twice/day ($T$=24.5 days): BED = 71.0 Gy;
- 30 × 2 Gy daily ($T$=42 days): BED = 73.1 Gy.

Thus, the BEDs of the three different schedules are not too different and—given an additional clonal heterogeneity and clonal selection by radiotherapy—may explain why the particular fractionation scheme had no significant effect on PFS or OS.

To the best of our knowledge, there was no favorable patient selection for HFRT protocols. Quite opposite, in one center (center 3) the HFRT scheme was preferably used in patients with involvement of or a tumor position close to the brain stem; this was also the case in some patients from center 1. Furthermore, some of the prognostic factors indicated a less favorable patient selection into the HFRT group: A significantly higher percentage of patients did receive concomitant steroids (76.1% versus 55.3%, Table 1), which is associated with poorer OS [52]. PTV sizes were also larger in the HFRT group, and a significantly lower percentage of patients received salvage therapy after tumor progression. For these reasons, we performed multivariable regression analysis with frailty terms in order to reduce any possible selection bias that is a general problem of retrospective analyses.

We were able to confirm the effect of several prognostic factors in univariable and multivariable analysis. The significant predictors were gross total versus non-gross total resection, *MGMT* hypermethylation, *IDH* mutation status, and salvage treatment. Mutations in the *IDH1 or IDH2* gene may predict a better prognosis by downregulating several hypoxia-inducible factor 1α (HIF-1α) target genes which are involved in glycolytic energy metabolism [53], among them lactate dehydrogenase (LDH) which catalyzes the conversion of pyruvate to lactate and thereby contributes to the acidification of the tumor microenvironment [54]. Accordingly, the LDH serum concentration was shown to be negatively correlated to OS in GB patients [33]. Both univariable and multivariable analyses also showed that OS was comparable between the HFRT and NFRT groups. However, there was a trend towards different PFS rates between the two groups, eventually pointing towards a systematic difference between centers in the evaluation of PFS within the scope of the RANO guidelines. The differentiation between pseudo-progression and real tumor regrowth is a difficult task in practice and our data reflect the real-world differences that exist between different applied criteria. In this respect, the difference in OS between institutions, which is an unambiguous endpoint, is harder to explain, but could be related to hospital-specific factors such as patient selection. For example, patients from center 3 had on average significantly larger PTVs than patients from the other institutions (all p < 0.0001). The negative selection was obvious in this patient cohort also by admission of HFRT in case of proximity to the brainstem. In addition, there might have been other unmeasured differences, so that the use of frailty models was a reasonable choice. This was confirmed by the importance of the frailty terms in multivariable modelling (Tables 3 and 4).

Another modality to reduce treatment time besides HFRT is hypofractionated accelerated radiation therapy. The use of hypofractionated protocols has been established in several randomized controlled trials [55–58]. This method is however primarily recommended for elderly patients and its role in the treatment of GB in younger individuals and those with good prognosis requires further research [59]. The other potentially interesting point of hypofractionation is the theoretical possibility of overcoming immanent or acquired radioresistance of tumor cells [44]. An alternative way of overcoming radioresistance could be a local dose escalation in biologically active tumor volumes, albeit the clinical data of dose escalation are needed to be proven in phase 3 trials [60]. Another possibility could be an intensification of chemotherapy regimens in selected patients which may also improve the treatment results with acceptable toxicity [61, 62].

Despite the large sample size, this study had several limitations. First, the presented data are retrospective in nature, which implies potential uncertainties in some variables, missing data, as well as the possibility of systematic confounding and biases. We took account of the problem of missing variables by using multiple imputation by chained equations, which is preferred over just using the data with complete cases because it makes better use of the full information within the data [45]. Sensitivity analysis with only the complete cases yielded quantitatively similar results as the imputed dataset analysis and therefore confirmed the latter, although uncertainties in regression coefficients were larger, as would be expected due to the smaller sample size. For example, *IDH* mutation status was a highly significant predictor of OS in multivariable regression on the imputed datasets but failed to reach the significance threshold in multivariable regression on the original dataset. Steroid administration was associated with worse OS in multivariable regression on both the imputed and original datasets but failed to reach the significance threshold. Nevertheless, the obtained hazard ratio > 1 is consistent with the detrimental effects of steroids found by Pitter et al. [52] which Klement and Champ [16] have attributed in part to the well-known effects of corticosteroids to raise blood glucose levels. In general, it is reassuring for the quality of our data that the hazard ratios of all variables are consistent with their expected effects on PFS and OS which are known from previous studies and tumor biology.

Another limitation is that the tolerability of the treatments was not directly evaluated because of the retrospective nature of this work. The need for steroids was assessed as an indicator of both tumor progression and treatment-related brain edema. Steroid administration was more frequent in the HFRT group. However, since prophylactic steroid administration was also performed, it cannot be considered an accurate surrogate parameter of toxicity. Previous data from Kaul et al. [39] and Lewitzki et al. [40] reported good tolerance of simultaneous TMZ and HFRT.

In conclusion, our multicenter analysis suggests that radiochemotherapy with HFRT and TMZ is a safe option for patients wishing to shorten their treatment time and does not affect OS, even in the context of larger tumors or poorer performance status. *MGMT* hypermethylation, smaller tumor size, and salvage therapy were confirmed to have the most significant positive impact on OS. To the best of our knowledge, both HFRT protocols were well tolerated without excessive acute or late toxicity, albeit no dedicated analysis was performed due to the retrospective nature. Analyses of neurocognition and quality of life in particular should also be performed in the future for a better assessment of tolerance. The rate of treatment interruptions was low in all protocol groups.

## Data Availability

The datasets generated during and/or analyzed during the current study are available from the corresponding author on reasonable request.

## Abbreviations

GB: Glioblastoma
HFRT: Hyperfractionated radiotherapy
NFRT: Normofractionated radiotherapy
PTV: Planning target volume
TMZ: Temozolomide

## Declarations

### Animal research statement

There was no animal research performed.

### Planned reproducibility statement

Our research group is planning a prospective observational trial to confirm the findings.

### Clinical trials registration

This research study was conducted retrospectively from data obtained for clinical purposes. No trial protocol and registration are applicable.
